# A full term abdominal pregnancy with an isthmic tubal implantation of the placenta

**DOI:** 10.1186/s12884-018-2071-z

**Published:** 2018-11-19

**Authors:** Sansan Rodrigue Sib, Issa Ouédraogo, Moussa Sanogo, Sibraogo Kiemtoré, Yobi Alexis Sawadogo, Hyacinthe Zamané, Blandine Bonané

**Affiliations:** 1 Ouahigouya University Teaching Hospital, Ouahigouya, BP 36 Burkina Faso; 2 Yalgado Ouédraogo University Teaching Hospital of Ouagadougou, 03 BP 7022, Ouagadougou, 03 Burkina Faso

**Keywords:** Abdominal pregnancy, Ectopic pregnancy, Placental implantation, Tubal isthmus

## Abstract

**Background:**

Abdominal pregnancy is defined as the partial or total insertion of the embryo into the abdominal cavity. It is rare, and can evolve towards the full term if it is not recognized in the early pregnancy. It carries a high risk of maternal-fetal morbidity and mortality.

**Case presentation:**

We report a case of a 22 year-old gravida IV, para II with an asymptomatic and undiagnosed abdominal pregnancy presumed full term, in a context of health centers under-equipment. She had attended 5 routine antenatal care, but had not performed any ultrasound scan. She had been transferred from a medical center to the Hospital of Ouahigouya (Burkina Faso) for bowel sub-obstruction and intrauterine fetal death, with failure of labor induction. On admission, the hypothesis of uterine rupture or abdominal pregnancy with antepartum fetal demise was considered. A laparotomy was then performed, where an abdominal pregnancy was discovered, and a dead term baby weighing 3300 g delivered. The placenta which was implanted into the ruptured isthmus of the left fallopian tube was removed by salpingectomy. Postoperative follow-up was uneventful.

**Conclusion:**

This case report exposes the necessity for the practitioner to think about the possibility of abdominal pregnancy in his clinical and sonographic practice, irrespective of the gestational age, mainly in contexts where there is under-equipment of the health centers.

## Background

Abdominal pregnancy is one of the topographic forms of ectopic pregnancy that is characterized by the partial or total implantation of the embryo into the abdominal cavity [1]. It is rare and carries a high risk of maternal and fetal morbidity and mortality when it is not recognized at the early stage of the pregnancy [1, 2]. Its evolution towards the term is seen most often in countries where the adapted means of diagnosis which are ultrasound, β-hCG measurement, and laparoscopy, are not very accessible [1, 2]. We report a case of full term abdominal pregnancy with an insertion of the placenta into the isthmic portion of the tube, discovered during a laparotomy indicated for uterine rupture or abdominal pregnancy.

## Case report

A 22-year-old woman, gravida 4, para 2, one early abortion and 2 alive children, was transferred from a medical center to the maternity ward of the University Teaching Hospital of Ouahigouya (Burkina Faso) for bowel sub-obstruction and intrauterine fetal death, with failure of labor induction, on an assumed full term pregnancy.

She first consulted for moderate abdominal pain that had been going on for 10 days, at the health center in her village, where early childbirth labor was diagnosed. The day after she arrived, she was evacuated to the referral medical center for fetal distress suggested by an abnormal decreasing of the fetal heart rate.

The history of the pregnancy notes that the patient, who did not know the exact date of her last menstrual period, had done in the health center of her village, 05 antenatal consultations during which no particular anomaly was noticed. The symphysio-fundal height grew steadily up to 30 cm at the last visit, with a presumed cephalic presentation. The patient did not perform any ultrasound or other blood tests outside the HIV serology that was negative. She did not experience pelvic pain or metrorrhagia at the beginning of her pregnancy, and never consulted for any pathology during her pregnancy. She had no particular medical and surgical history.

On admission at the maternity ward of the University Teaching Hospital of Ouahigouya, the patient no longer complained of abdominal pain, but reported respiratory discomfort due to abdominal distension, and absent fetal movements.

She had normal hemodynamic state, but mild pallor. Her abdomen was distended, and the fetal parts were palpated under the maternal abdominal wall, with difficulty in specifying the presentation. The sounds of the fetal heart were not perceived. At the vaginal touch, the cervix was anterior, short, soft, and dehiscent, and the fingerstall was stained with traces of blood. There was no ileus.

Diagnostic hypothesis of uterine rupture or abdominal pregnancy with intrauterine fetal death were thought about.

Emergency ultrasound scan noted an empty uterus of subnormal size, a fetal death and a lateral uterine mass reminding the placenta.

**Fig. 1:**
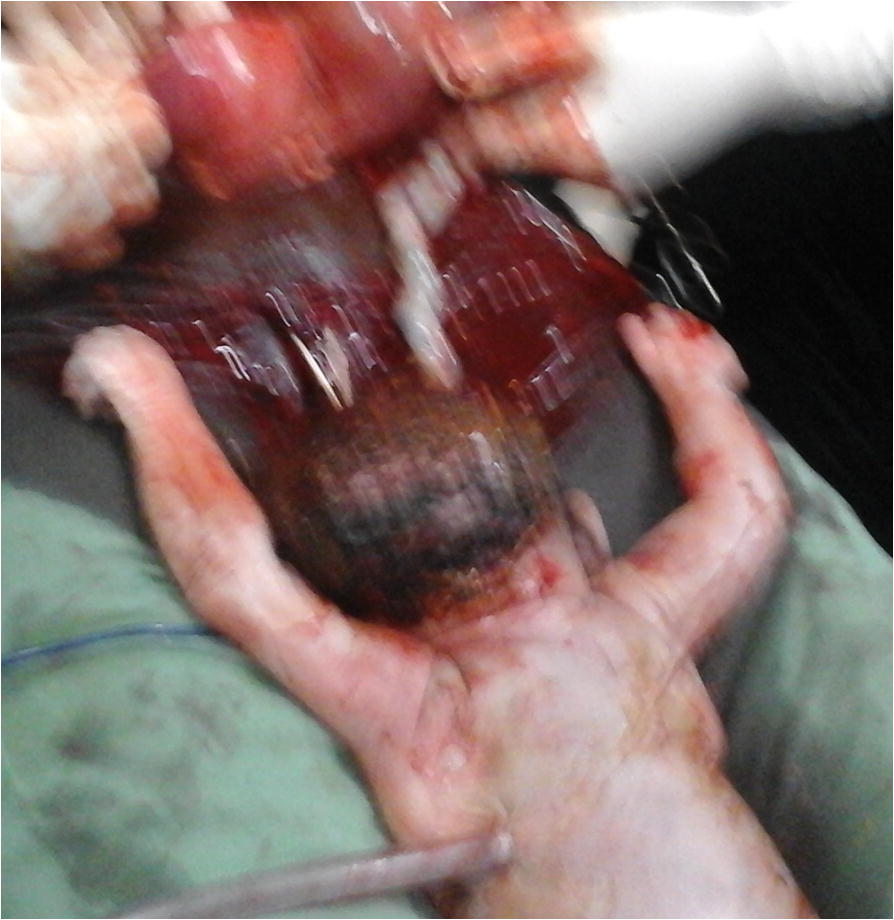
Dead new born extracted from the abdominal cavity

**Fig. 2:**
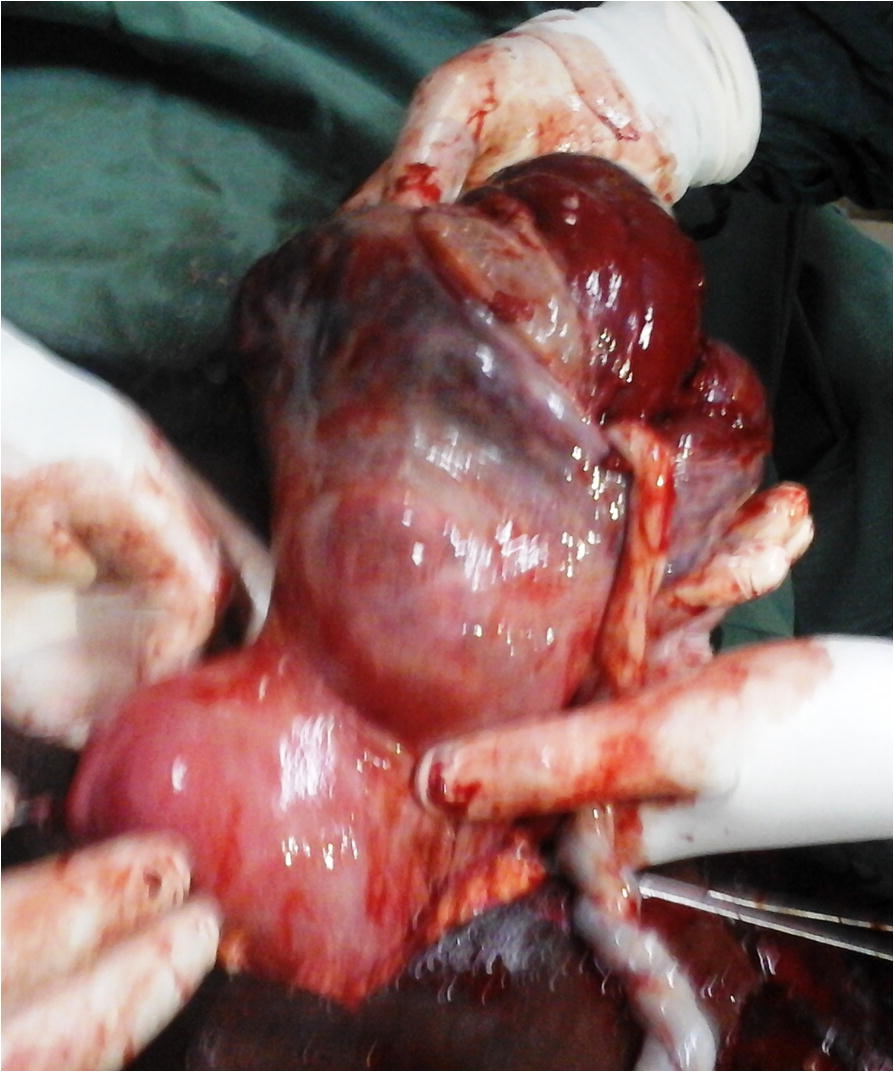
Hypertrophied and ruptured isthmus with the placenta within it

**Fig. 3:**
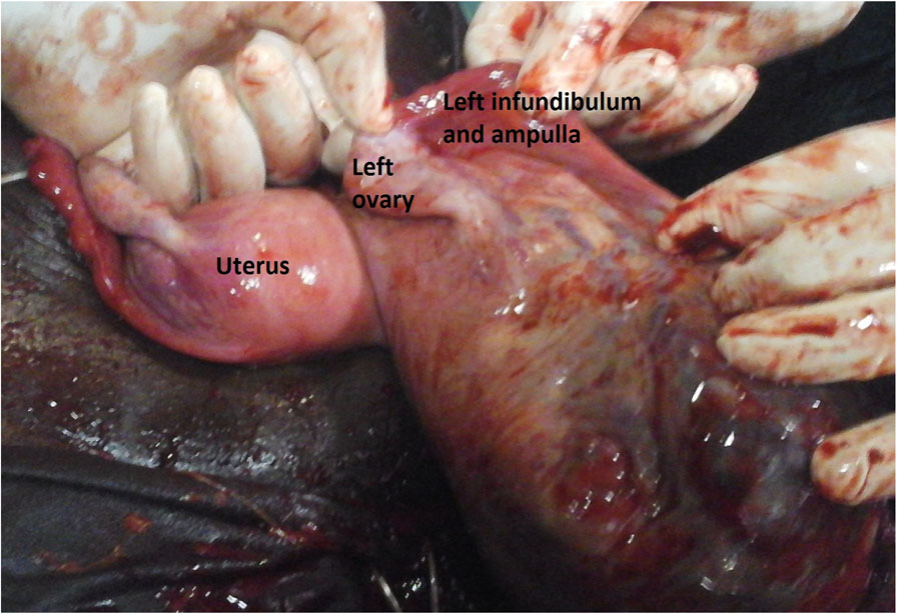
Left ovary, ampullary and infundibular left portions following the isthmus

**Fig. 4:**
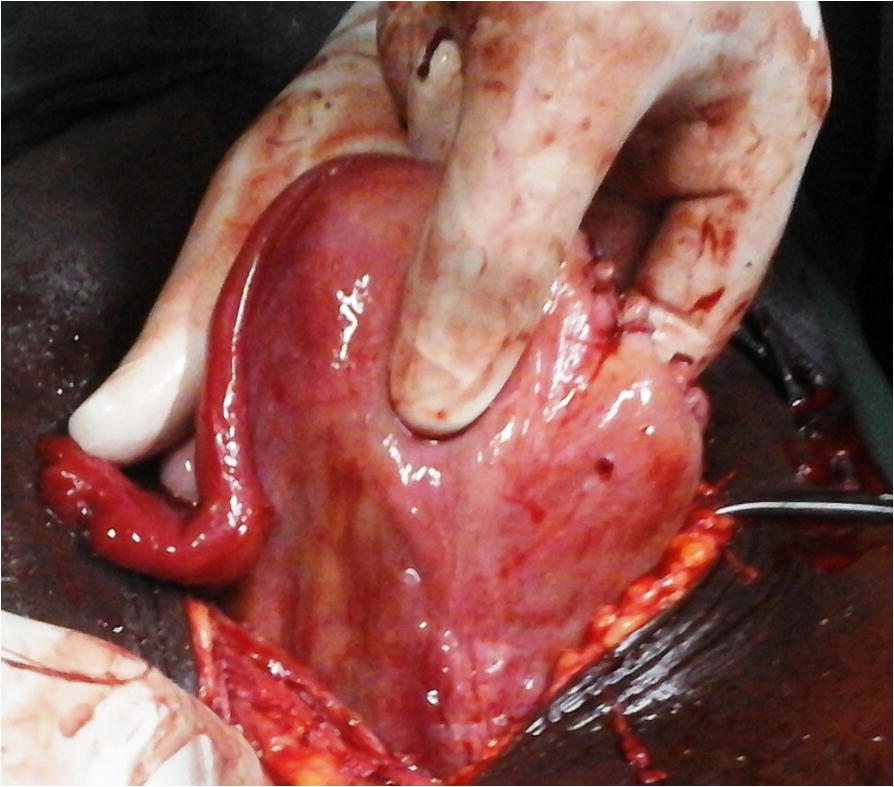
Appearance of the uterus after the salpingectomy

An emergency laparotomy was then indicated for suspicion of abdominal pregnancy or uterine rupture. At the opening of the peritoneum, a bloody fluid (most likely blood, amniotic fluid and peritoneal fluid) was sucked up. The fetus that bathed in the abdominal cavity was extracted dead and macerated (Fig. 1). It weighed 3300 g and did not have any malformation. The placenta was fully inserted into the hypertrophied and ruptured isthmus of the left fallopian tube (Fig. 2). The ipsilateral ampullary and infundibular portions were free and normal-looking (Fig. 3). The uterus was non gravid and had normal size. The abdominal viscera were free from adherence. The diagnosis of full term abdominal pregnancy, with implantation of the placenta into the isthmus of the left tube was then retained. A left total salpingectomy removing in the same time the placenta (Fig. 4) was performed. The patient received a blood transfusion, and the postoperative follow-up was simple.

## Discussion

### Epidemiology

Abdominal pregnancy is rare. It represents approximately 1% of ectopic pregnancies [3] which incidence reaches 2% [4]. The abdominal pregnancy’s incidence seems to be greater in developing countries where sexually transmitted diseases with tubal lesions would be common [1, 2, 5]. Its evolution towards the term seems also to be more frequent in these same countries poorly equipped and medicalized [1, 2].

### Pathogenesis

The implantation of the embryo in the abdominal cavity can be primitive or secondary. It is primitive in case of direct implantation of the embryo in the abdominal cavity. It is secondary when it occurs after a ruptured tubal pregnancy or a tubal abortion or even a uterine rupture or perforation [3, 5]. Secondary implantation is the most common [1, 6, 7]. In our case the abdominal location is secondary because the embryo was first inserted into the tubal isthmus, as shown by the implantation of the placenta into this part of the tube. The development of the fetus took place in the abdominal cavity, through the wall of the ruptured isthmus. The diameter of the isthmus is small, 2 mm [8], so its rupture probably occurred early, at least in the first trimester [6].

### Diagnosis

The diagnosis of abdominal pregnancy is difficult [3, 5], and is an intra-operative finding in 40 to 50% of cases [3, 5], despite antenatal follow-up and ultrasound scan. The evolution of abdominal pregnancy when it is asymptomatic [3, 9], as in our case, raises the question of whether the clinical examinations were carried out appropriately. One would expect signs of hemoperitoneum and shock, as part of the tubal rupture leading to secondary abdominal implantation. Their absence could in our case be explained by the fact that the embryo, part of which (placenta) was still intratubal, herniated through the ruptured tubal wall, has caused a hemostasis by compression of the vessels of the rupture banks. For Worley et al. [6] the clinical expression of abdominal pregnancy is variable, depending on the degree of the anatomical distortion it creates and the placental insertion site.

Clinical signs are therefore not specific, but for some authors [1–3, 5, 6], the following signs should bring the practitioner to think about the diagnosis: abdominal pain with intestinal transit disorder, abdominal pain during active movements of the fetus, spreading of the abdomen due to an irregular presentation, palpation of the fetal parts under the maternal abdominal wall, cervix deviated under pubis symphysis, failure to trigger childbirth labor. Unfortunately most of these signs only appear during already advanced abdominal pregnancies, as with our patient.

The ultrasound scan which was not done in our case because of the under-equipment of our health centers, should be done early in the first trimester, to specify the location of the pregnancy and at the same time the correct gestational age [4]. At this time of pregnancy, the risk of missing the diagnosis of ectopic pregnancy is lower [4, 9]. For best results, it is better to do this transvaginally, paying particular attention to the ovaries and the uterine wall [3]. In the second and third trimesters, the risk of misrecognizing abdominal pregnancy is important, and has reached more than 50% in some series [7, 9]. For Allibone et al. [10] absence of uterine wall between maternal bladder and fetus, ectopic location of placenta, abnormal presentation of fetus, proximity of fetal parts to maternal abdominal wall and absence of amniotic fluid between placenta and the fetus, are the ultrasound features of abdominal pregnancy.

Measurement of serum β-hCG is also used in the diagnosis of early ectopic pregnancies. When an initial measurement is performed, a threshold of more than 1500 IU / L associated with an empty uterus on endovaginal ultrasound should suggest an ectopic pregnancy [8]. Serial measurements of β-hCG levels can also be performed in patients with possible ectopic pregnancy. For Heather et al. and Surampundi et al. these measures may have been elevated, sometimes as in an intrauterine pregnancy, in decline or level of β-hCG levels, and therefore require caution in their interpretation [11, 12] which should be associated with the clinical and ultrasound results to achieve a correct diagnosis [12]. In case of persistent doubt despite ultrasound and measurement of β-hCG, MRI can be used to clarify the diagnosis [6].

### Treatment and prognosis

The treatment of abdominal pregnancy is surgical, at best by laparotomy, for a better control of the hemorrhagic risk related to the extraction of the placenta [1, 5, 7]. This extraction is justified only if it is easily achievable, with a lower hemorrhagic risk [2, 5]. Otherwise the placenta left in place will gradually and spontaneously resorb in the post operative course. The use of methotrexate to accelerate this resorption is controversial for it would involve a greater risk of infection due to an accelerated placental necrosis [3, 5, 6].

In our case, after fetal extraction, we performed a total left salpingectomy which allowed removing the placenta in the same time. An attempt at placental detachment would have been hemorrhagic and would have laminated the tube, the preservation of which might have given rise to another ectopic pregnancy later. For many authors [1, 9, 13], the ablation of the placenta is associated with a better maternal prognosis, as in our case. When the placenta is left in place, it is necessary to keep watch over the appearance of the following maternal complications in postoperative period: bowel obstruction, infection, hemorrhage, anemia, fistula, [1, 9, 13] etc. These complications can worsen the maternal prognosis, with a lethality up to 18% [1, 5, 7]. The fetal prognosis varies with the gestational age. When the diagnosis is early, before 20 weeks [14], it is unlikely that the pregnancy will be preserved [3, 14]. At a later age, a rigorous monitoring can allow the extraction of the live newborn as soon as the period of fetal viability is reached [3]. Otherwise, when the diagnosis is late, or when it is done intra-operatively, the fetal prognosis is often very pessimistic, like in our case, with a perinatal mortality which varies between 40 and 95% according to authors [1, 5, 6, 9, 13].

## Conclusion

Abdominal pregnancy is a situation of high risk of morbidity and mortality for the fetus and the mother. Its diagnosis is difficult so careful examination of the pregnant woman is important, looking for any suspicious sign of ectopic pregnancy. The health authorities of our developing countries should make an effort to make routine early ultrasound accessible to pregnant women, and the obstetricians should keep in mind the possibility of ectopic pregnancy, irrespective of the gestational age [1, 3, 13].

## Abbreviation

MRI: Magnetic resonance imaging
β-hCG: Beta human chorionic gonadotropin

## References

[1] Mahi M, Boumdin H, Chaouir S, Salaheddine T, Attioui D, Amil T (2002). A new case of abdominal pregnancy. J Radiol.

[2] Aliyu LD, Ashimi AO (2013). A multicentre study of advanced abdominal pregnancy: a review of six cases in low resource settings. Eur J Obstet Gynecol Reprod Biol.

[3] Faller E, Kauffmann E, Chevrière S, Heisert M, Ranjatoelina H, Boumahni B (2006). Full term abdominal pregnancy. J Gynecol Obstet Biol Reprod.

[4] Cohen, L, Diagnostic ultrasound in the first trimester of pregnancy. Glob Libr Women's Med., (ISSN: 1756–2228) 2017; Doi 10.3843/glowm.10094

[5] Riethmuller D, Courtois L, Maillet R, Schaal JP (2003). Ectopic pregnancy management: cervical and abdominal pregnancies. J Gynecol Obstet Biol Reprod.

[6] Worley KC, Hnat MD, Cunningham FG (2008). Advanced extrauterine pregnancy: diagnostic and therapeutic challenges. Am J Obstet Gynecol.

[7] Bang Ntamack JA, Ngou Mve Ngou JP, Sima Ole B, Sima Zue S, Mayi Tsonga S, Meye JF (2012). Abdominal pregnancy in Libreville from 1999 to 2009. J Gynecol Obstet Biol Reprod.

[8] Lansac J, Lecomte P, Marret H (2012). Gynécologie pour le praticien. 8^ème^ edition.

[9] Nassali MN, Benti TM, Bandani-Ntsabele M, Musinguzi E (2016). A case report of an asymptomatic late term abdominal pregnancy with a live birth at 41 weeks of gestation. BMC Res Notes.

[10] Allibone GW, Fagan CJ, Porter SC (1981). The sonographic features of intra-abdominal pregnancy. J Clin Ultrasound.

[11] Murray H, Baakdah H, Bardell T, Tulandi T (2005). Diagnosis and treatment of ectopic pregnancy. Can Med Assoc J.

[12] Surampudi K, Gundabattula SR (2016). The role of serum Beta hCG in early diagnosis and management strategy of ectopic pregnancy. J Clin Diagn Res.

[13] Sunday-Adeoye I, Twomey D, Egwuatu EV, Okonta VI (2011). A 30-year review of advanced abdominal pregnancy at the mater Misericordiae hospital, Afikpo, southeastern Nigeria (1976–2006). Arch Gynecol Obstet.

[14] Poole A, Haas D, Magann EF (2012). Early abdominal ectopic pregnancies: a systematic review of the literature. Gynecol Obstet Investig.

